# Supplementary material for: Fruit and Vegetable Consumption and Their Polyphenol Content Are Inversely Associated with Sleep Duration: Prospective Associations from the UK Women’s Cohort Study
Source: Nutrients. 2018 Nov 20;10(11):1803. doi: 10.3390/nu10111803 (PMC6266198; doi:10.3390/nu10111803)
Supplement: Supplementary file 1 [file nutrients-10-01803-s001.pdf]

ACTIVITY

37. On an average **weekday** how is your day spent?

	**Number of hours & / or minutes in a 24 hour day spent doing the following activities?**	
	Hours	Minutes
Sleeping		
Sitting		
Light activities (e.g. washing, dressing, eating)		
Standing		
Household chores (e.g. vacuuming, ironing)		
Lifting heavy objects		
Light exercise (e.g. walking, yoga, easy gardening)		
Moderate exercise (e.g. fast walking, easy swimming, hill walking, easy cycling)		
Strenuous exercise (e.g. running, vigorous swimming, high impact aerobics)		

38. On an average **weekend day** how is your day spent?

	**Number of hours & / or minutes in a 24 hour day spent doing the following activities?**	
	Hours	Minutes
Sleeping		
Sitting		
Light activities (e.g. washing, dressing, eating)		
Standing		
Household chores (e.g. vacuuming, ironing)		
Lifting heavy objects		
Light exercise (e.g. walking, yoga, easy gardening)		
Moderate exercise (e.g. fast walking, easy swimming, hill walking, easy cycling)		
Strenuous exercise (e.g. running, vigorous swimming, high impact aerobics)		

Figure S1. Sleep duration assessment method.

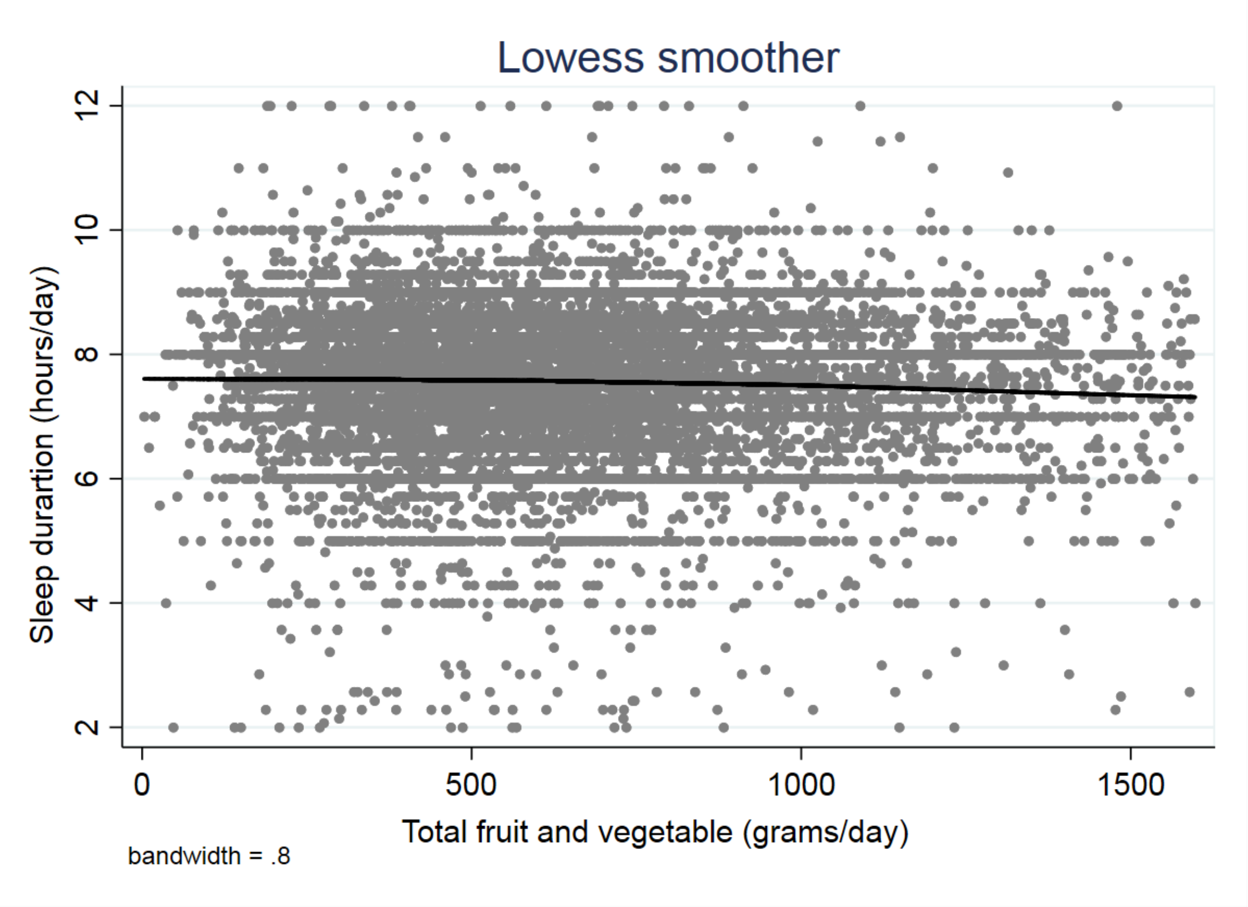

Figure S2. The relationship between fruit and vegetable intakes and sleep duration using locally weighted scatterplot smoothing (LOWESS).

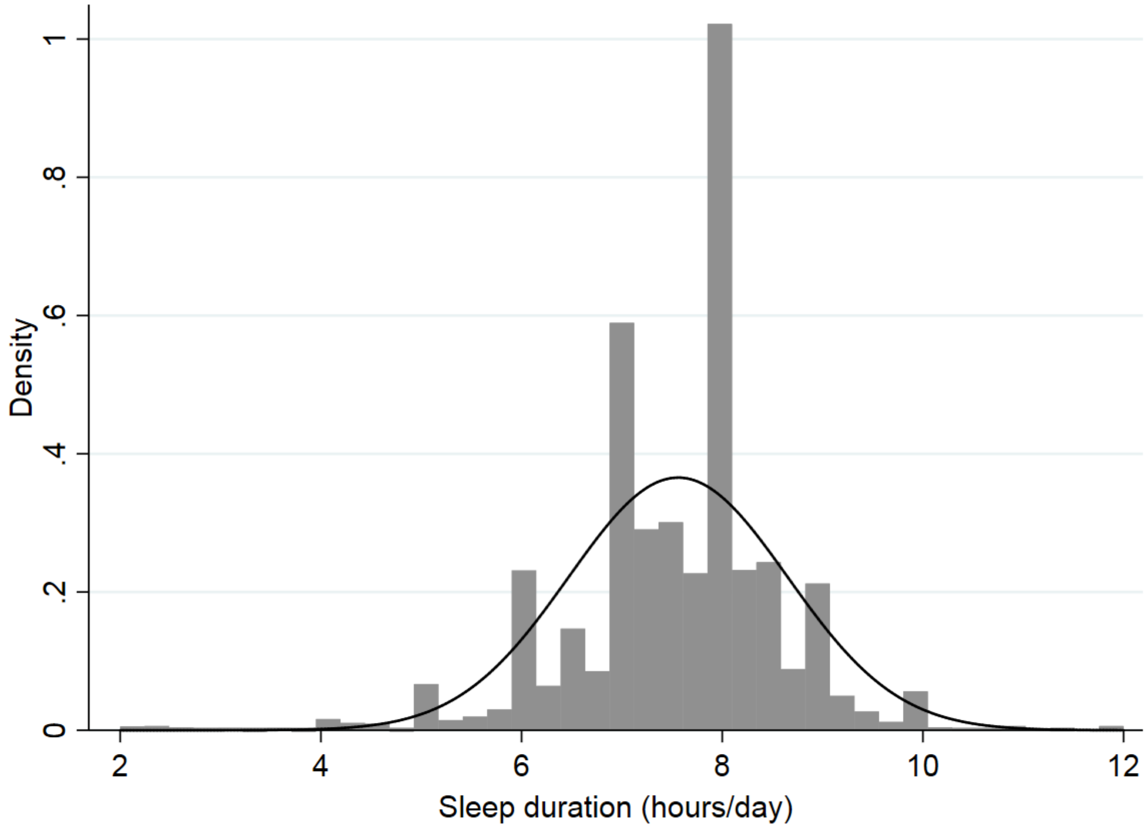

Figure S3. Normal distribution of sleep duration (outcome) using histogram plot.
